# Prey naïveté rather than enemy release dominates the relation of an invasive spider toward a native predator

**DOI:** 10.1002/ece3.7905

**Published:** 2021-07-13

**Authors:** Nijat Narimanov, Kamal Hatamli, Martin H. Entling

**Affiliations:** ^1^ iES Landau Institute for Environmental Sciences Department of Ecosystem Analysis University of Koblenz‐Landau Landau Germany

**Keywords:** Araneae, biological invasions, enemy release hypothesis, invasion biology, Linyphiidae, *Mermessus trilobatus*

## Abstract

Ecosystems may suffer from the impact of invasive species. Thus, understanding the mechanisms contributing to successful invasions is fundamental for limiting the effects of invasive species. Most intuitive, the enemy release hypothesis predicts that invasive species might be more successful in the exotic range than resident sympatric species owing to the absence of coevolution with native enemies. Here, we test the enemy release hypothesis for the invasion of Europe by the North American spider *Mermessus trilobatus*. We compare the susceptibility of invasive *Mermessus trilobatus* and a native species with similar life history to a shared predator with which both species commonly co‐occur in Europe. Contrary to our expectations, invasive *Mermessus trilobatus* were consumed three times more frequently by native predators than their native counterparts. Our study shows that invasive *Mermessus trilobatus* is more sensitive to a dominant native predator than local sympatric species. This suggests that the relation between the invasive spider and its native predator is dominated by prey naïveté rather than enemy release. Further studies investigating evolutionary and ecological processes behind the invasion success of *Mermessus trilobatus*, including testing natural parasites and rapid reproduction, are needed to explain its invasion success in Europe.

## Graphical abstract

Most intuitive, the enemy release hypothesis predicts that invasive species might be more successful in the exotic range than resident sympatric species owing to the absence of coevolution with native enemies. Here, we, for the first time, test the enemy release hypothesis for the invasion of Europe by the North American spider *Mermessus trilobatus*. Contrary to our expectations, invasive spiders were consumed three times more frequently by native predators than their native counterparts, suggesting that the relation between the invasive spider and its native predator is dominated by prey naïveté rather than enemy release.

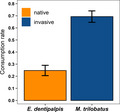

## 1 INTRODUCTION

Nonindigenous species can play influential roles in their exotic range once becoming invasive. Invasions are considered successful when alien species establish and rapidly expand their ranges in novel environments by overcoming biogeographical barriers and ecological pressures (Sakai et al., 2001). The impact of invasive species on native ecosystems has been described since the middle of the 20th century (Elton, 1958). However, the mechanisms behind the often striking success of invasive species are still uncertain (Schultheis et al., 2015). Up to 39 hypotheses were developed to better describe the processes behind successful invasions (Enders et al., 2019). As one of the most predominant and intuitive, the enemy release hypothesis posits that nonindigenous species are released from the pressure of predators and parasites once introduced to their exotic ranges (Elton, 1958; Keane & Crawley, 2002). Introduced species might have advantages over resident species against native enemies by, for example, not being recognized as prey or hosts for parasites in the exotic range (e.g., Cottrell & Shapiro‐Ilan, 2003; Gozzi et al., 2020; Montes et al., 2020; Tierney et al., 2020). However, Elton (1958) mentions that species leaving coevolved predators and parasites from the native areas immediately meet novel potential enemies once introduced in the exotic range. Hence, due to the lack of the coevolutionary history with novel predators, parasitoids, and pathogens, introduced species might be naïve toward novel enemies' archetypes under comparable or even higher enemy pressure in their exotic range (Cox & Lima, 2006).

Despite the popularity of the enemy release hypothesis (Hierro et al., 2005), the growing literature provides only mixed support (Heger & Jeschke, 2014; Schultheis et al., 2015). Such a discrepancy might come from the studies' different approaches based on the scale of the analysis. Biogeographical studies investigate enemy release comparing invasive animals from native and exotic populations. In contrast, community studies examine native and invasive species from the same community in the exotic range (Colautti et al., 2004). While studies at the biogeographical scale largely support the enemy release hypothesis, the results from community studies are equivocal (Colautti et al., 2004). Such differences between biogeographical and community scale studies might arise due to, for example, failure to distinguish two types of enemy release, namely compensatory, when the limited resources utilized for defense are repositioned elsewhere, and regulatory, when the loss of enemies leads directly to increase in demographic parameters (Colautti et al., 2004). This may lead to inaccurate conclusions about the net effect of enemy release at biogeographical scales. Further, studies investigating enemy release comparing the number of enemy species between natural and exotic ranges at the biogeographical scale might be ambiguous since invasive species and their enemies are often better studied in their native rather than exotic range. Hence, more enemies would be expected in native populations due to sampling efforts (Colautti et al., 2004). Additionally, only a portion of the population is being relocated to exotic regions during the transport of invasive species. Therefore, introduced populations are often genetically less diverse compared with native populations. Hence, such invasion bottlenecks could also lead to nonvalid comparisons of the populations on the biogeographical scale (for a more extensive review, see Colautti et al., 2004). Consequently, all introduced species lose enemies at the biogeographical level, irrespective of their release from enemies in their introduced range at the community level (Colautti et al., 2004).

An increasing number of studies indicate a changing role of enemy release through the different invasion phases, namely introduction, establishment, and spread (first: Drake, 2003; reviewed in Roy et al., 2011). Accordingly, release from enemies might play different roles during the introduction, establishment, or spread of invasive species in their exotic range. For instance, the parasitism of invasive mosquito *Aedes albopictus* (Diptera: Culicidae) by a native enemy is low in the introduced area only for at least two years following the colonization (Aliabadi & Juliano, 2002). Still, many invasive species fail to establish in the exotic regions after introduction. One of the most plausible contributing mechanisms of establishment failure of invasive species may be an increased pressure by novel enemies in the introduced range (Cox & Lima, 2006; Elton, 1958). Therefore, the enemy release hypothesis as a driving force behind successful invasions should be tested for already established invasive species that are in their spreading phase (i.e., abundant or dominant) in the exotic range. Furthermore, studies investigating the role of enemy release as a causal mechanism of invasiveness are mainly based on invasive plant and vertebrate species (e.g., Carpenter & Cappuccino, 2005; Gozzi et al., 2020; Hawkes, 2007; Hierro et al., 2005; van Kleunen et al., 2010; Lankau et al., 2004; Liu & Stiling, 2006; Meijer et al., 2016; Montes et al., 2020; Schultheis et al., 2015; Tierney et al., 2020), whereas only a limited number is focused on arthropods (e.g., Aliabadi & Juliano, 2002; Juliano et al., 2010; Paula et al., 2021; Zuharah & Lester, 2010).

Spiders play essential roles in ecosystems (Birkhofer et al., 2017; Michalko et al., 2019) and can consume up to 800 million tons of prey annually (Nyffeler & Birkhofer, 2017). Despite the growing dominance both in agricultural (e.g., Hogg & Daane, 2010) and natural habitats (e.g., Pétillon et al., 2020), invasive spiders have only recently started to attract scientific attention (Campbell et al., 2020; Narimanov et al., 2021; Nentwig, 2015). The role of enemy release behind successful spider invasions, to our knowledge, has never been tested.

The North American dwarf spider *Mermessus trilobatus* (Araneae: Linyphiidae; formerly known as *Eperigone trilobata*; Millidge, 1987) was first recorded in Europe in the late 1970s near Karlsruhe in South‐West Germany (Dumpert & Platen, 1985). The species has undergone a concentric range expansion in Europe, spreading by > 1,000 km in less than 50 years (Hirna, 2017) and often reaching high local abundances (Narimanov et al., 2021; Schmidt et al., 2008). The invasion success of *M*. *trilobatus* seems to be neither based on high competitiveness toward native linyphiids (Eichenberger et al., 2009) nor a ruderal strategy, as they do not benefit from soil disturbance (Narimanov et al., 2021). Therefore, reduced susceptibility to native predators might explain the invasion success of *M*. *trilobatus* in Europe.

Here, we investigate, at the community level, whether the invasion success of *M*. *trilobatus* in Europe is explained by the release from the pressure of native predators. Thus, we compare the invasive *M*. *trilobatus* and a native sympatric species' susceptibility to a shared native predator with which they frequently co‐occur. We expect that in contrast to the shared coevolutionary history of the native prey and predator, invasive *M*. *trilobatus* would benefit from reduced predation by native predators, which could explain their invasion success in Europe.

## 2 MATERIAL AND METHODS

### 2.1 Study species

We chose *Erigone dentipalpis* (Araneae: Linyphiidae) as native prey because of their similar size (Table 1) and hunting mode to *M*. *trilobatus* and because the two species often dominate in the same habitats (Narimanov et al., 2021). Spiders are exposed to various natural enemies, including other spiders as perhaps the most important predators (Foelix, 2011). Therefore, we chose *Pachygnatha degeeri* (Araneae: Tetragnathidae; body length = 3–4.2 mm), the most abundant linyphiid‐eating spiders, as predators for the experiments. *Pachygnatha degeeri* are free hunters living close to the ground of the grasslands where both *M*. *trilobatus* and *E*. *dentipalpis* are found and can easily climb and invade linyphiid webs. Moreover, these generalist predators are not found in North America and, thus, are ideal candidates as native European predators (Nentwig et al., 2021). We sampled all spiders from perennial hay meadows as the preferred habitat of *M*. *trilobatus* (Narimanov et al., 2021). The meadows were situated next to the river Queich, close to Landau in Germany (see Table 2 for coordinates). Spiders were sampled between July and September 2020 using a vacuum sampler (modified STIHL SH86 blower; Stihl, Waiblingen, Germany). We sampled 14 *M*. *trilobatus* and 16 *E*. *dentipalpis* females and 85 adult *P*. *degeeri* individuals. All linyphiids were transferred individually into glass jars (405 ml) with a 1 cm layer of moist plaster of Paris to ensure high humidity inside the glasses. We kept all spiders in climate cabinets under standard conditions (25℃, RH = ~65%, L:D = 16:8). We fed all linyphiids *ad libitum* with springtails (*Sinella curviseta*) to obtain a high number of egg sacs. We transferred all offspring singly into 30‐ml glass jars with a layer of humid plaster on the bottom and fed ad libitum until adulthood. We also kept all *P*. *degeeri* individuals in 100‐ml glass jars with a layer of humid plaster on the bottom and fed *ad libitum* with drosophila flies (*Drosophila hydei*).

**TABLE 1:** Mean values of prosoma widths (in mm), sample sizes, and the status for Europe of all spider species used for the experiments

Species	Family	Status	Prosoma widths (mm)					
			Females		Males		Combined	
			Mean	*N*	Mean	*N*	Mean	*N*
*Erigone dentipalpis*	Linyphiidae	Native	0.75	55	0.84	46	0.795	101
*Mermessus trilobatus*	Linyphiidae	Invasive	0.68	55	0.73	46	0.701	101
*Pachygnatha degeeri*	Tetragnathidae	Native	1.21	38	1.09	47	1.144	85

Note

Spider names follow the World Spider Catalog (Nentwig et al., 2021).

**TABLE 2:** Geographical coordinates of six grassland sites where all spider species (invasive *Mermessus trilobatus* and native *Erigone dentipalpis* and *Pachygnatha degeeri*) were collected

Site	Latitude	Longitude	Location
1	N49°12′03.0″	E8°08′49.2″	Landau
2	N49°12′10.7″	E8°09′24.8″	Landau
3	N49°12′16.2″	E8°06′22.3″	Landau
4	N49°12′02.7″	E8°08′57.6″	Landau
5	N49°12′05.8″	E8°10′11.4″	Offenbach an der Queich
6	N49°11′59.7″	E8°09′18.3″	Landau

### 2.2 Experimental design

Experiments were performed in 405‐ml glass jars with approximately 1 cm layer of moistened plaster on the bottom and five vertical sticks to facilitate web building. We used only adult linyphiids reared in the laboratory. Prior to experiments, we measured all spiders' prosoma widths (see Table 1 for means) as an estimate of body size that is independent of the current feeding condition (Moya‐Laraño et al., 2008). We assigned a randomly chosen pair of prey (invasive *M*. *trilobatus* and native *E*. *dentipalpis*) to the same predator (*P*. *degeeri*). Then, each linyphiid pair was tested with the same predator during two trials in random order. We calculated the difference in prosoma width by subtracting the respective value of the predator minus the prey. We let linyphiids build webs in the glasses without food for two days before experiments. All linyphiids, irrespective of sex and species, built a web. Simultaneously, we starved predators also for two days prior to each trial for standardization. From previous observations, we expected that *E*. *dentipalpis* builds the web slightly closer to the surface than *M*. *trilobatus*. As spiders in low webs may be more exposed to ground‐hunting predators such as *P*. *degeeri*, we sprayed the webs with water and measured their lowest and the highest position to the plaster in each glass after two days of web building. We placed predators on the surface of the plaster, avoiding any damage to webs, and gave the trials three days. We checked spiders every 24 hr. In total, we had 202 trials and tested 101 *M*. *trilobatus* and 101 *E*. *dentipalpis*.

### 2.3 Statistical analysis

We modeled the consumption rate (consumed, not consumed) by fitting a generalized linear mixed‐effect model (GLMM) for a binomial response from the lme4 package (Bates et al., 2015) in R 4.0.3 (R Core Team, 2020). We then applied ANOVA chi‐square test (the car package in R; Fox & Weisberg, 2019) to the GLMM model to analyze the effects of prey species (*M*. *trilobatus, E. dentipalpis*), prey and predator sex, the difference in prosoma width of the predator and prey, and web minimum and maximum positions to the surface on the consumption rate. We included predator ID as a random factor since each predator was used at least twice during experiments. Additionally, we modeled the linyphiids' web positions to the surface (minimum and maximum) by fitting linear models (lm) from the R package stats (R Core Team, 2020) and included linyphiid species as fixed predictors. We then applied ANOVA *F* test to the lm models to investigate the web‐building strategies of two species (*M*. *trilobatus* and *E*. *dentipalpis*). We validated the lm model results using permutation tests (PermTest function from pgirmess package in R; Giraudoux, 2021).

## 3 RESULTS

**FIGURE 1:**
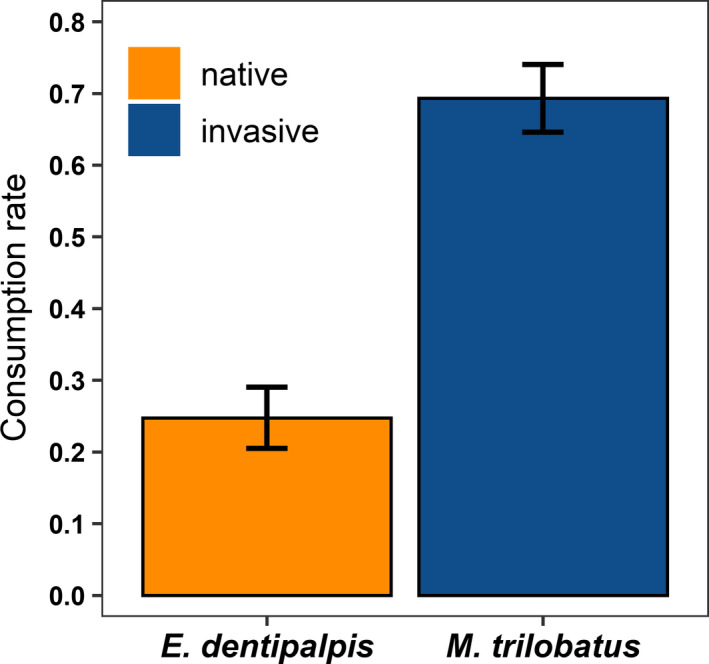
Effects of prey susceptibility to predators based on prey species (invasive: *Mermessus trilobatus*; native: *Erigone dentipalpis*). Means ± *SE* are presented

**FIGURE 2:**
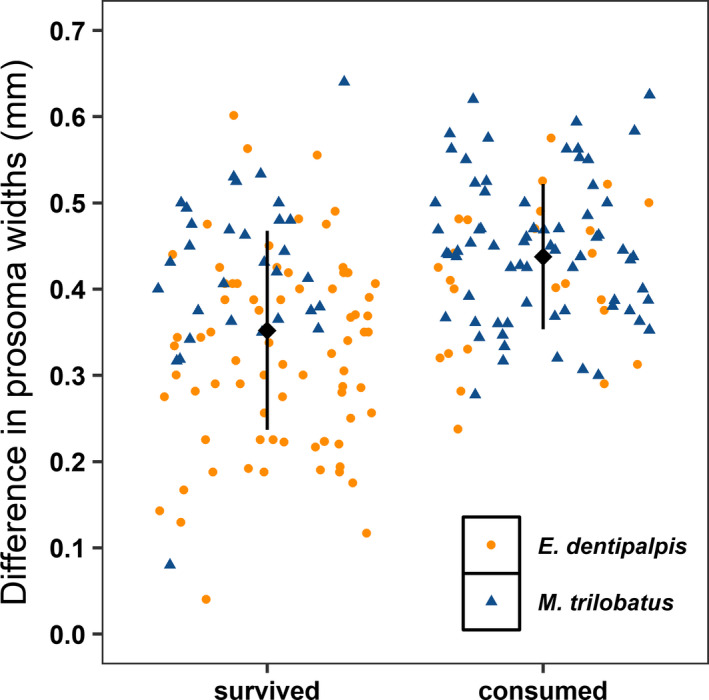
Effects of the difference in prosoma width between predator and prey on prey survival. Invasive and native prey species are shown with different shape and color (blue triangles for invasive *Mermessus trilobatus* and orange dots for native *Erigone dentipalpis*). Means ± *SE* are presented

**FIGURE 3:**
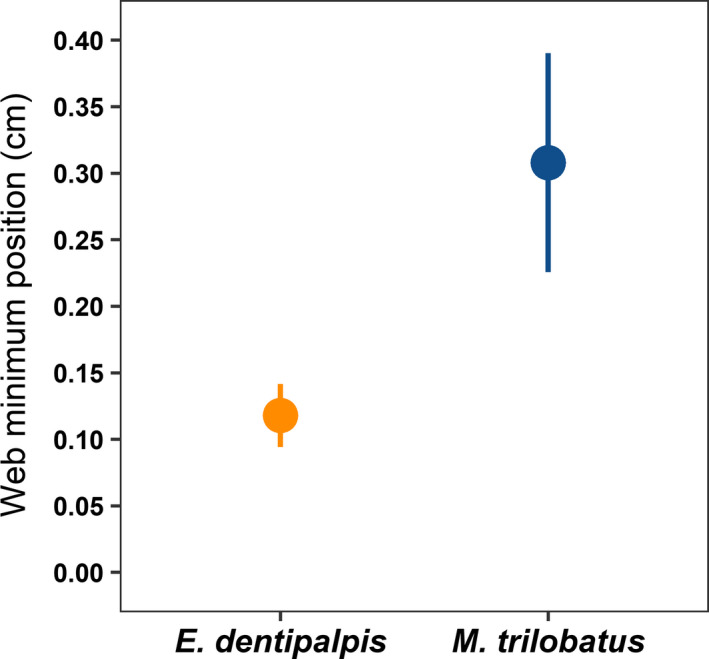
Effects of linyphiids' web minimum positions (distance to the surface in cm) based on species (native *Erigone dentipalpis* and invasive *Mermessus trilobatus*). Means ± *SE* are presented

Opposite to our expectation, the invasive *M*. *trilobatus* was consumed almost three times more often compared with native *E*. *dentipalpis* (Table 3, Figure 1). Furthermore, smaller prey (compared with predators) were consumed with slightly a higher rate than larger ones (Table 3, Figure 2). There were no effects of spiders' sex (predators and prey) and linyphiids' web positions (minimum and maximum) on their susceptibility to predation (Table 3). However, on average, native *E*. *dentipalpis* built their webs around 2.5 times closer to the surface (plaster) than invasive *M*. *trilobatus* (web minimum; *F*_1, 200_ = 9.843, *p* = .002; Figure 3). There was no difference in linyphiids' web maximum positions to the surface (web maximum; *F*_1, 200_ = 2.472, *p* = .118). In total, 95 out of 202 linyphiids were consumed. The highest number of *M*. *trilobatus* was consumed during the first two days (35 and 24, respectively), followed by the last day (11). Similarly, the highest number of *E*. *dentipalpis* (15) was consumed the first day, leaving the following two days with an equal number of individuals consumed (5 each). A higher number of females of *E*. *dentipalpis* compared with males were consumed (20 and 5, respectively), whereas similar numbers of *M*. *trilobatus* females and males were consumed during experiments (39 and 31, respectively).

**TABLE 3:** Outputs for logistic regression model predicting the consumption rate of prey by their species, the difference in prosoma widths between predators and prey (calculated by subtracting the respective value of the predators minus prey), spiders' sex (predators and prey), and the distance of the web to the surface (web minimum and maximum)

	*χ^2^*	*p*
Prey species	12.49	.**0004**
Difference in prosoma widths	5.57	.**018**
Sex of prey	0.41	.52
Sex of predators	0.27	.603
Web minimum	0.16	.693
Web maximum	0.09	.761

Note

Significant correlations are shown in bold.

## 4 DISCUSSION

To our knowledge, this is the first empirical study testing the enemy release hypothesis on a spider. Contrary to our expectations, invasive *M*. *trilobatus* was more susceptible to native European predators than a sympatric native species. Our results support that the consumption rate is dependent on the predator–prey size difference (e.g., Binz et al., 2014; Preisser & Orrock, 2012). With increasing size differences, prey were consumed more frequently. Moreover, as expected, *E*. *dentipalpis* built the webs closer to the ground surface than invasive *M*. *trilobatus*. Nevertheless, web positions (minimum and maximum) had no significant effects on linyphiids' consumption rate by predators during our trials.

During our experiments, native predators consumed seventy individuals of invasive *M*. *trilobatus* compared with only 25 native *E*. *dentipalpis*, indicating possible invasive prey naïveté toward local predators. The prey naïveté hypothesis predicts that native prey often fail to recognize and/or avoid an introduced predator due to a lack of the coevolutionary history (Cox & Lima, 2006; Sih et al., 2010). Similarly, but in a different scenario where introduced species are prey for local predators, lack of native predator recognition by invasive prey can result in increased predation on the introduced species (e.g., Barrio et al., 2010; Carthey & Banks, 2018; Ruland & Jeschke, 2020). We observed that *M*. *trilobatus* frequently abandoned their webs after the predators' intrusion, and once outside their webs were easily subdued by *Pachygnatha*. In contrast, native *E*. *dentipalpis* usually remained sheltered in their webs during our experiments. *Pachygnatha degeeri* were unable to reach *E*. *dentipalpis* hiding in the densest parts of their webs (KH and NN, personal observations). Thus, native linyphiids might have a better surviving strategy against these local predators through hiding rather than fleeing. Suppose such behavior is an adaptation to the situation in Europe. In that case, this could be directly tested using spiders from North America, where *E*. *dentipalpis* is invasive in the native range of *M*. *trilobatus* and confronted with North American generalist predators, which are not found in Europe (e.g., *Pachygnatha autumnalis* or *P. brevis*; World Spider Catalog, 2021).

Our results show that the invasive *M*. *trilobatus* is sensitive toward local European predators. *Mermessus trilobatus* has undergone rapid concentric range expansion in Europe. Spiders used in our experiments were derived from the populations less than 50 km of the presumed core of the invasion range (Dumpert & Platen, 1985). Individuals in these areas were present for at least 45 years, during which local predators might have adapted to these novel prey. Indeed, a meta‐analysis by Hawkes (2007) found that invasive plant species may accumulate novel enemies over time. Additionally, another meta‐analysis by Chun et al. (2010) showed that invasive plant species suffered relatively less damage than native species studied in the fields compared with greenhouses. Hence, some invasive species may dominate in the fields where natural enemies do not recognize them as a suitable food source, but these enemies would feed on them in enclosed conditions (e.g., Siemann & Rogers, 2003; Lankau et al., 2004; Siemann et al., 2006; but see Carpenter & Cappuccino, 2005). Consequently, ecological and evolutionary processes that drive invasions might change over time (Hawkes, 2007) and different phases of the invasion process (Drake, 2003; Roy et al., 2011), whereby local predators may increasingly recognize invasive species as potential prey over time. However, most studies on the enemy release hypothesis are focused on invasive plants and vertebrates and only a limited number on arthropods. Therefore, studies investigating enemy release as a causal effect of successful arthropod invasion and the loss of enemy pressure over time are needed to bridge this research gap. Investigations comparing susceptibility of *M*. *trilobatus* to *P*. *degeeri* derived from the populations where *M*. *trilobatus* have never been found (e.g., Ireland, Russia) could test for a possible loss of enemy release and/or adaptation of *P*. *degeeri* to *M*. *trilobatus* as novel prey over time.

The invasive *M*. *trilobatus* tested here are sensitive toward native predators, indicating possible high top‐down controlled systems. Indeed, a recent meta‐analysis showed that spiders' total biomass across 54 North American grasslands failed to increase with total invertebrate biomass (Welti et al., 2020), indicating the potential control by their own predators (Sanders & Platner, 2007). Nevertheless, *M*. *trilobatus* was successful in colonizing a major part of Europe in a relatively short time. Higher reproductive ability of *M*. *trilobatus* compared with native sympatric species, balancing their high sensitivity to local predators, might still explain their rapid colonization success in Europe.

The invasion success of *M*. *trilobatus* is not explained by the ruderal strategy (Narimanov et al., 2021), and laboratory experiments showed that *M*. *trilobatus* are less competitive than native linyphiids (Eichenberger et al., 2009). Additionally, our results suggest that invasive *M*. *trilobatus* is more susceptible to local predators, namely *P*. *degeeri,* compared with a native linyphiid species. Yet, it is possible that release from parasitoids and pathogens may play a role in the colonization success of *M*. *trilobatus* in Europe. This deserves further investigation. Further, *M*. *trilobatus* might spread in their invasion range without being pressured by interspecific interactions, coexisting with natural species in the same habitats (Narimanov et al., 2021).

In conclusion, our study finds no evidence for the enemy release from a generalist native predator of the invasive *Mermessus trilobatus* in Europe. On the contrary, the invasive spiders were consumed at higher rates than native sympatric species, likely due to their naïveté toward resident predators. Previous studies investigating mechanisms behind the colonization success of *M*. *trilobatus* in Europe also found no evidence for the role of soil disturbance (Narimanov et al., 2021) or higher competitive ability toward local sympatric species (Eichenberger et al., 2009). Therefore, other potential mechanisms behind their rapid spread and successful establishment, notably their high reproduction, remain to be investigated.

## CONFLICT OF INTEREST

None declared.

## AUTHOR CONTRIBUTIONS

**Nijat Narimanov:** Conceptualization (supporting); data curation (equal); formal analysis (lead); investigation (supporting); methodology (equal); visualization (lead); writing—original draft (lead); writing—review and editing (lead). **Kamal Hatamli:** Data curation (supporting); investigation (lead); methodology (supporting); writing—original draft (supporting); writing—review and editing (supporting). **Martin H. Entling:** Conceptualization (lead); Data curation (equal); formal analysis (supporting); funding acquisition (lead); investigation (supporting); methodology (equal); supervision (lead), visualization (supporting); writing—original draft (supporting); writing—review and editing (supporting).

## Data Availability

The dataset generated and analyzed during this study is available in the *Figshare* repository [https://doi.org/10.6084/m9.figshare.14500239.v1].
